# Effects of the IAA-Producing Endophytic *Bacillus* spp. on the Growth of *Hordeum vulgare* L.

**DOI:** 10.3390/microorganisms14051069

**Published:** 2026-05-09

**Authors:** Murat Güler

**Affiliations:** Ministry of National Education, Kırsehir 40100, Türkiye; volvox2015@gmail.com

**Keywords:** barley, endophytic bacteria, *Bacillus mycoides*, *Bacillus tropicus*

## Abstract

Endophytic bacteria are beneficial microbes that live within plant tissues and promote growth through nitrogen fixation, phosphate solubilization, and phytohormone production. Two endophytic isolates from bell pepper (*Capsicum annuum* L.) root were identified based on their morphology and biochemical properties using 16S rRNA gene sequencing. Winter barley seeds were inoculated with two PGP (plant growth-promoting) bacterial strains (C-14 and C-27), previously characterized for indole-derived compound (IDC) production, and evaluated in a pot experiment with four treatments: Treatment A1 (C-14), Treatment A2 (C-27), Treatment A3 Consortium (C-14 + C-27), and Treatment A4 (non-inoculated control). The results indicated that root and stem lengths increased in plants inoculated with bacteria compared to the uninoculated control. Among treatments, A2 produced the greatest root and shoot lengths (17.23 and 26.2 cm), while A3 showed the lowest (15.8 and 21.5 cm). SPAD values also increased by 6%, 10%, and 3.2% in Treatments A1, A2, and A3, respectively. This study clearly demonstrated that the endophytic isolates (C-14 and C-27) obtained from bell pepper roots significantly enhanced the growth of barley due to their ability of IDC production, thereby offering a promising alternate to chemical fertilizers.

## 1. Introduction

Barley (*Hordeum vulgare* L.) is a fundamental cereal crop widely cultivated across the globe for its dual use as human food, in beer production, and its use as animal feed. Its high dietary fiber content, especially its abundance of β-glucan, has been associated with numerous health-promoting effects, including the prevention of degenerative diseases such as diabetes, obesity, hypertension, and colitis [1]. In addition to health benefits, barley is valued for its rich nutritional profile, serving as a cheap source of high-quality starch, vitamins, minerals, and proteins, which enhances its potential as a dietary supplement. Despite its agronomic and nutritional significance, barley production like many other crops is increasingly affected by various environmental and anthropogenic stressors [2]. The reduction in cultivable land, frequent crop losses, global warming, prolonged drought, and natural disasters have all contributed to substantial yield declines. In this context, achieving agricultural sustainability necessitates not only improvements in the physical and chemical characteristics of the soil but also the conservation and enhancement of its biological properties. Promoting soil health through environmentally friendly practices is essential. The widespread use of synthetic fertilizers and pesticides poses significant risks to soil ecosystems. Thus, the implementation of green production methods that prevent soil and water pollution has become a strategic priority everywhere [3].

Endophytes constitute a diverse group of microorganisms including primarily bacteria and fungi, along with archaea, algae, protozoa, and viruses, that live on or close to various plant organs such as roots, stems, leaves, seeds, and fruits. The microorganisms must initially colonize the rhizosphere, followed by successful invasion of the plant endosphere to establish themselves as endophytes. This process relies on specific microbial traits, including motility, surface attachment, degradation of plant polymers, and the ability to evade plant defense mechanisms [4]. Endophytic bacteria colonize internal plant tissues and support plant growth by numerous methods such as nitrogen fixation, phosphate solubilization, and the synthesis of siderophores and phytohormones. Additionally, these bacteria produce enzymes like ACC (1-aminocyclopropane-1-carboxylate) deaminase, which reduces ethylene levels in plants, thereby enhancing plant growth [5]. These bacteria can synthesize a variety of IDCs (indole-derived compounds) [6]. Indoles and their derivatives such as indole-3-acetic acid (IAA), indole-3-butyric acid (IBA), indole-3-carboxaldehyde (ICA), indole-3-hexanoic acid (IHA), and indole-3-acetonitrile (IAN) are organoheterocyclic compounds widely used as plant growth regulators in agricultural systems [7]. Among these, IAA is the principal biologically active phytohormone, while other indole compounds predominantly function as intermediates or metabolites in its biosynthesis [8].

Endophytic bacteria are increasingly recognized as integral components of sustainable agriculture due to their ability to establish intimate, non-pathogenic associations within plant tissues. In cereal crops such as barley (*Hordeum vulgare* L.), these microorganisms enhance plant performance by modulating physiological processes through improved nutrient acquisition, phytohormone production, and increased tolerance to both abiotic and biotic stresses [9].

Although research on the plant growth-promoting effects of endophytic bacteria has increased in recent years, studies specifically focusing on their impact on barley remain limited; nevertheless, existing evidence indicates that endophytic members of the genera *Bacillus*, *Pseudomonas*, and *Azospirillum* can significantly enhance barley performance by improving root system architecture, increasing nitrogen availability, and promoting photosynthetic efficiency, highlighting the need to further investigate the potential of indigenous isolates across different ecosystems and plant species [10,11]. In particular, although *Bacillus* is among the most frequently isolated genera in barley and other cereal crops, studies specifically evaluating the growth-promoting effects of *Bacillus mycoides* and *Bacillus tropicus* on barley remain limited [12].

In addition, endophytic bacteria contribute to plant health by producing antimicrobial compounds and inducing systemic resistance, thereby reducing the impact of soil-borne pathogens [13]. Owing to these multifunctional roles, they have gained increasing importance in the context of climate change and the growing demand for environmentally sustainable agricultural practices. Consequently, the use of endophytic bacteria as bioinoculants represents a promising strategy to reduce reliance on chemical fertilizers while maintaining or enhancing barley productivity [14,15].

This study aimed to assess the plant growth-promoting traits of endophytic bacteria isolated from bell pepper and to examine the effects of two bacterial strains (*Bacillus mycoides* C-14 and *Bacillus tropicus* C-27) on barley growth under controlled pot conditions.

## 2. Materials and Methods

### 2.1. Sample Collection

**Figure 1:**
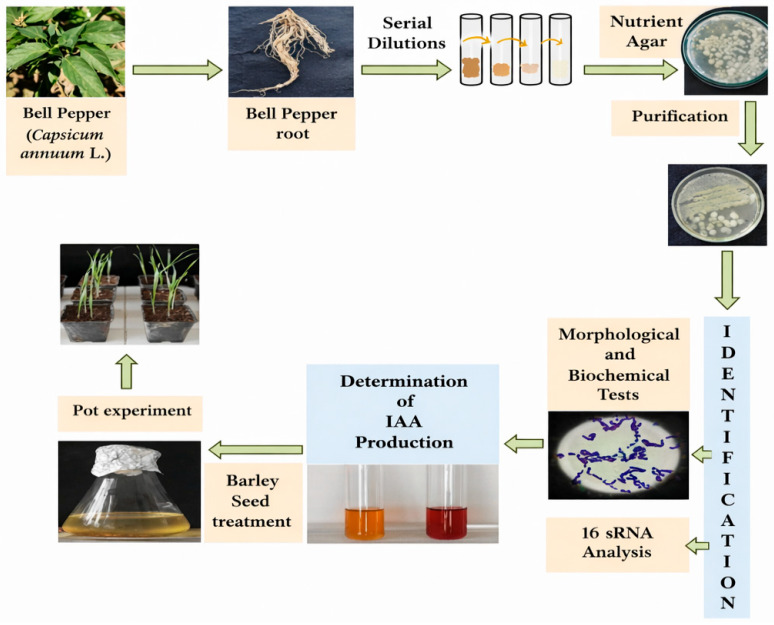
Graphical abstract illustrating the effects of endophytic bacteria (C-14 and C-27), isolated from bell pepper, on the growth and development of barley plants.

Although the bacterial isolates were obtained from bell pepper tissues, barley was intentionally selected as the experimental plant to assess the cross-host plant growth-promoting potential of these endophytic strains. Previous studies have demonstrated that endophytic and rhizobacterial microorganisms can colonize and promote growth in non-native host plants, indicating that their beneficial effects are not strictly limited to their original host [16,17]. Moreover, barley is a well-established model cereal frequently used in plant–bacteria interaction studies due to its agronomic relevance and experimental suitability. Thus, its use in this study enables a more comprehensive evaluation of the functional potential and broader applicability of the tested bacterial isolates. Bell pepper (*Capsicum annuum* L. var. grossum) was obtained from a bell pepper field in Çayağzı (Cemele) village (39°16′56.0″ N, 34°04′44.3″ E), Kırsehir, in May 2023. The pepper plants were properly labeled, sealed in sterile plastic bags, and transported to the bacteriology laboratory of the Department of Field Crops, Faculty of Agriculture, Ankara University, under cold-chain conditions for subsequent isolation procedures (Figure 1).

### 2.2. Isolation of Endophytic Bacterial Isolates

Potential endophytic bacterial strains were isolated from the roots of three bell peppers following the method described by Amaresan et al. [11]. Bell pepper roots were washed thoroughly in tap water for 30 min to remove soil and similar particles. The roots were cut into 2–3 cm lengths, placed in a beaker and washed 3–4 times in sterile distilled water. The cut roots were washed with 70% ethanol for 25 s, and then rinsed 5–6 times with sterile distilled water for 5 min. Surface-disinfected root tissues were crushed and ground aseptically with homogenizers. The homogenized plant tissues were further macerated in sterile phosphate-buffered saline (0.2 g KCl, 8 g NaCl, 1.44 g Na_2_HPO_4_, 0.24 g KH_2_PO_4_, in 1 L of distilled water, pH 7.4) and subsequently subjected to serial dilution up to 10^−3^. From each dilution, 100 µL was spread on to Nutrient Agar (NA) plates and incubated at 30 °C for 48 h. After incubation, bacteria were examined and selected based on desired morphological characteristics and then they were purified by the streak plate method. Purified bacteria were stored in 50% (*v*/*v*) glycerol solution at −80 °C for future research.

Figure 1 illustrates the flowchart of the process used to identify endophytic bacteria isolated from bell pepper roots to determine their growth on the barley.

### 2.3. Phenotypic/Biochemical Characterization and Molecular Identification

#### 2.3.1. Morphological and Biochemical Characteristics

Morphological and biochemical characteristics of the bacterial isolates, including Gram staining, endospore formation, catalase activity, and oxidase activity, were assessed according to the protocols described by Palleroni et al. [18].

#### 2.3.2. Molecular Identification of Bacterial Endophytes

##### DNA Extraction

Genomic DNA was extracted from two endophytic isolates (C-14 and C-27) using the GeneMATRIX Bacterial & Yeast DNA Isolation Kit (EurX^®^, Przyrodnikow 3, Gdanjsk, Poland) following the manufacturer’s instructions (https://eurx.com.pl/docs/manuals/en/e3580.pdf, accessed on 4 February 2025). The DNA quantity and purity were evaluated following extraction using a NanoDrop 2000 spectrophotometer (Thermo Scientific, Waltham, MA, USA, USA).

##### PCR Amplification of 16S rRNA and Sequencing

Amplification of the 16S rRNA gene was performed using universal primers BAC 27F (5′ AGAGTTTGATCMTGGCTCAG 3′) and BAC 1492R (5′ TACGGYTACCTTGTTACGACTT 3′) along with the FIREPol^®^ DNA Polymerase Kit (Solis Biodyne Company, Tartu, Estonia) in a 25 μL reaction volume [19]. PCR was conducted in a Kyratec thermocycler with the following conditions: 95 °C for 5 min for initial denaturation, followed by 30 cycles of denaturation at 95 °C for 45 s, annealing at 57 °C for 45 s, and extension at 72 °C for 60 s, with a final extension at 72 °C for 5 min. The amplified product, approximately 1470 bp, was run on a 1.5% agarose gel prepared with 1× TAE buffer. Electrophoresis was performed at 100 volts for 90 min, and the results were visualized under UV light using ethidium bromide staining. Single-band PCR products were purified using the HighPrep™ PCR Clean-up System (AC-60005, MAGBIO (MagBio Genomics Inc. 200 Professional Dr. Gaithersburg, MD 20879, USA)) according to the manufacturer’s protocol. Purified 16S rDNA amplicons were sequenced using the Sanger dideoxy sequencing method with an ABI 3730XL Sanger sequencing instrument (Applied Biosystems, Foster City, CA, USA) and the BigDye Terminator v3.1 Cycle Sequencing Kit (Applied Biosystems, Foster City, CA, USA).

##### Bioinformatics Analysis

The resulting sequences, including two chromatograms for each bacterium (forward and reverse), were visualized, checked for quality, and analyzed using the Finch TV program version 1.4.0. They were then transformed into consensus sequences using Clustal W2 (clustalw-mac-universal-2.0.tgz) (http://www.ebi.ac.uk/Tools/msa/clustalo/, accessed on 4 February 2025). Similarities and identities of the query sequences were determined using the nucleotide Basic Local Alignment Search Tool (BLASTn (BLASTDBv5); https://blast.ncbi.nlm.nih.gov/, accessed on 4 February 2025) and the rRNA/ITS GenBank database of NCBI. The sequences were submitted to the GenBank nucleotide database. For constructing the phylogenetic tree, highly similar sequences were subjected to multiple sequence alignment (MSA) using ClustalW2 (clustalw-mac-universal-2.0.tgz) in BioEdit 7.7 software. The maximum likelihood (ML) phylogenetic tree was constructed using Kimura’s 2-parameter substitution model. The bootstrap method was applied with 1000 replicates. Evolutionary analyses were performed using Molecular Evolutionary Genetics Analysis Version 7.0 (MEGA7) software [20].

### 2.4. Characterization of IDC-Producing Endophytic Bacteria

#### 2.4.1. Qualitative Estimation of IDC Production

IDC production by the isolates was assessed following the method of Etesami et al. [21]. Bacterial cultures were incubated under pure conditions for 48 h at 36 ± 2 °C. After incubation, the cultures were centrifuged at 10,000 rpm for 10 min to obtain the supernatant. An aliquot of 2 mL of the supernatant was then combined with two drops of orthophosphoric acid and 4 mL of Salkowski reagent (50 mL of 35% perchloric acid and 1 mL of 0.5 M FeCl_3_ solution). The suspension was incubated in dark conditions for 30 min and the development of a pink coloration was taken as evidence of IDC production.

#### 2.4.2. Quantitative Estimation of the IDC Production Capacity

The production of IDC was assessed in cultures grown in nutrient broth (NB) medium supplemented with 0.2% L-tryptophan, incubated at 30 °C with shaking at 200 rpm for 4 days. One milliliter of culture was sampled every 24 h and stored at −20 °C for analysis. Following the method outlined by Gang et al. [22], 100 µL of Salkowaski reagent (FeCl_3_, 0.5 M; HClO_4_, 35%) was combined with an equal volume (100 µL) of the culture supernatant (obtained by centrifuging at 10,000 rpm for 5 min) in a 96-well microplate. The mixture was incubated in the dark at room temperature for 30 min. After the color developed, the absorbance of the mixture was measured spectrophotometrically (SHIMADZU UVmini-1240 Spectrophotometer) at 530 nm using both a standard curve created from known IDC concentrations and an uninoculated NB medium as controls. Optical density (OD) was recorded daily at 530 nm until the fourth day.

### 2.5. Seed Preparation and Bacterial Inoculation

The winter barley seeds (*Hordeum vulgare* L.) used in this study were obtained from the Department of Field Crops of the Faculty of Agriculture, Ankara University (39°57′44.2″ N, 32°51′36.7″ E). Two endophytic isolates with plant growth-promoting properties were used as barley seeds treatments. Barley seeds were placed in 10% sodium hypochlorite (NaOCl) solution for 15 min. This was followed by rinsing five times with distilled tap water to ensure sterilization. Then, the samples were air-dried in laminar air flow. The pure cultures were grown on nutrient broth (NB) medium containing 4% sugar (sucrose). For bacterial application, two isolates were first cultured in 200 mL nutrient broth for 48 h at 28 °C in 250 mL Erlenmeyer flasks sealed with Parafilm. The concentration of the prepared bacterial solutions (including 4% sugar) was adjusted to 10^8^ cfu ml^−1^ with sterile distilled water. The seeds were then treated with bacterial suspensions for 45 min under sterile conditions. Following the protocol of Heinonsalo et al. [23], control seeds underwent the same sterilization process but were treated with sterile distilled water instead of bacterial inoculum.

### 2.6. Greenhouse Pot Trials

A pot experiment was conducted to study the effect of two endophytic isolates (C-14 and C-27) with IAA-producing properties on the growth of barley plants. The plants were grown for 30 days in climate cabinets under controlled conditions of 20 °C temperature, 65% relative humidity, and 10 h fixed light photoperiod with light intensity (850 PAR), followed by 14 h of complete darkness [24]. The experiment was conducted in Complete Randomized Design (CRD) with three replicates.

#### 2.6.1. Growth Medium and Soil Properties

Pots (4.5 cm × 4.5 cm × 10 cm) were sterilized with potassium permanganate using garden soil:vermiculite:perlite as a growth medium (1:1:1 ratio). The physicochemical profile of the soil are detailed in Table 1. The electrical conductivity (ECe), pH, organic matter content, and total N, P, and K of the soil extract were analyzed according to the methods described by Bao et al. [25]. The soil organic matter content was determined using the K dichromate volumetric method. The available N, P, and K were determined using the diffusion absorption method, the molybdenum–antimony colorimetric method, and ammonium acetate extraction–flame spectrophotometry, respectively.

The garden soil samples to be used in pots were sterilized by autoclaving at 121 °C and 1.1 atm pressure. Single colonies of each isolate were individually inoculated into NB medium and cultured under shaking conditions at 28 °C with horizontal shaking at 200 rpm for 24 h. The bacterial suspensions were subsequently adjusted to a final concentration of 1 × 10^8^ cfu ml^−1^ and utilized in further experiments. All treatments are described as follows:

Treatment A1: Seeds bacterized with C-14;

Treatment A2: Seeds bacterized with C-27;

Treatment A3: Seeds bacterized with consortium (C-14 + C-27);

Treatment A4: Seeds of barley plants coated with 1% CMC (carboxymethylcellulose) slurry served as control.

A total of 5 seeds were sown in each of the 12 pots, with 3 replicates per treatment. The inoculated treatments included 20 mL of the bacterial suspension at a concentration of 1 × 10^8^ cfu ml^−1^ into each pot. The uninoculated control treatments (A4) contained 20 mL of sterile Luria–Bertani (LB) liquid medium. Sterile water was slowly added to each pot every three days to maintain soil moisture. The physicochemical characteristics of the soil employed in the experiment are presented in Table 1.

#### 2.6.2. Data Collection

After 30 days, the plants were harvested to evaluate plant fresh weight, dry weight, root length, stem length, and relative chlorophyll content (SPAD units). Total chlorophyll content in barley leaves was determined using a SPAD chlorophyll meter (SPAD-502, Minolta, Osaka, Japan). Measurements were taken from different leaves of the seedlings, with five replicates per treatment.

### 2.7. Statistical Analysis

Statistical analyses of the data were performed using JMP Pro 17.0 software based on three replicates. The normality of the data was assessed, and variables with normal distribution were expressed as mean ± standard deviation (SD). Differences among treatments were determined by analysis of variance (ANOVA) followed by Duncan’s multiple range test (*p* ≤ 0.05).

## 3. Results

### 3.1. Morphological and Biochemical Characterization

In the current study, two endophytic bacteria were identified based on morphological observations and biochemical characterization. Physiological and biochemical characteristics, along with Gram staining of the bacterial isolates, were assessed according to the protocols described by Palleroni et al. [18]. Based on the phenotypic characterization of the two isolates, both were determined to be Gram-positive with the ability to form spores, exhibited motility, and tested positive for the oxidase test; however, isolate C-27 was found to be catalase-negative. Details are presented in Table 2.

### 3.2. Molecular Identification of Strains C-14 and C-27

**Figure 2:**
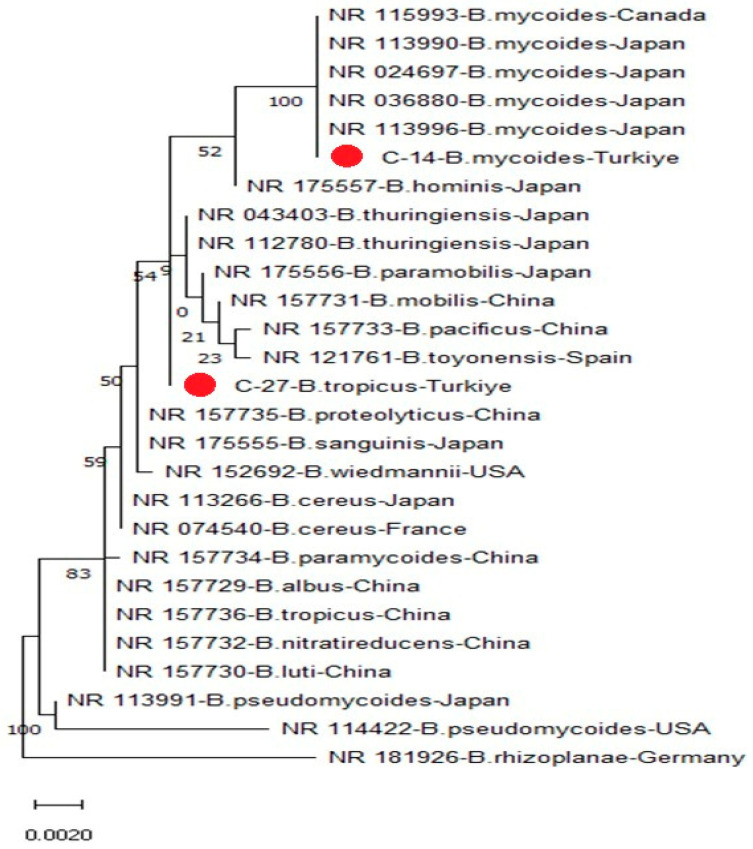
Maximum likelihood phylogenetic tree of *B. mycoides* strain C-14 and *B. tropicus* strain C-27 based on the 16S rRNA gene.

The 16S rRNA gene sequences of isolates C-14 and C-27 were analyzed using in silico tools and have been deposited in the NCBI GenBank under accession numbers PQ610418 and PQ610419, respectively (Table 3). Isolate C-14 exhibited 99.93% identity with reference strains of Bacillus mycoides isolated from various countries (NR_113996, NR_036880, NR_024697, NR_115993, and NR_113990), while isolate C-27 showed 99.72% identity with Bacillus tropicus strain MCCC 1A01406 (NR_157736). The phylogenetic tree shows that C-14 clusters with B. mycoides strains, whereas C-27 forms a separate clade (Figure 2).

### 3.3. Evaluation of IDC Production

**Figure 3:**
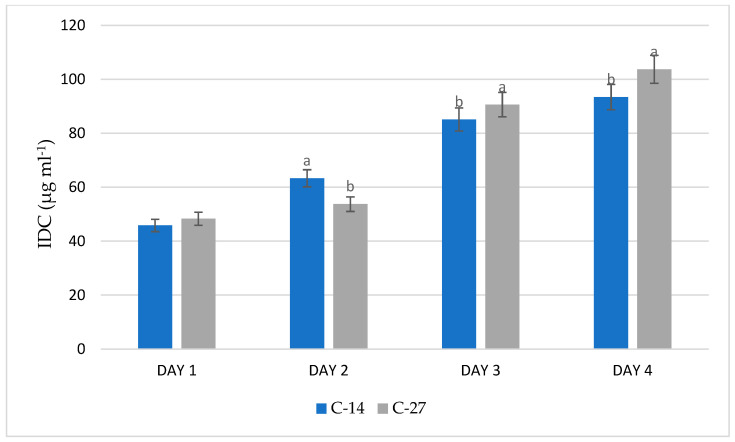
Single-panel bar chart showing indole-3-carbinol (IDC) production by endophytic bacterial strains C-14 and C-27 cultured in nutrient broth supplemented with 0.2% L-tryptophan over a 4-day incubation period. Blue and gray bars represent strains C-14 and C-27, respectively. Data are expressed as mean ± standard deviation (SD) of three independent replicates (*n* = 3). Different lowercase letters indicate statistically significant differences between treatments within each incubation day according to one-way ANOVA followed by LSD post hoc test (*p* ≤ 0.05).

The C-14 and C-27 isolates exhibited a pink coloration, indicating their capacity for indole-derived compound (IDC) production. In this study, both strains produced IDC at different quantitative levels when grown in nutrient broth (NB) supplemented with L-tryptophan. Spectrophotometric analysis showed variation in IDC production between the two isolates at each sampling day. On day 4, the highest IDC production was recorded in isolate C-27 (103.7 µg mL^−1^), followed by isolate C-14 (93.4 µg mL^−1^). Within each sampling day, C-27 generally showed higher IDC values than C-14. The lowest IDC production was observed in isolate C-14 on day 1 (45.8 µg mL^−1^), while the highest value was observed in isolate C-27 on day 4 (103.7 µg mL^−1^) (Figure 3). Statistical analyses were performed separately for each incubation day; therefore, comparisons are valid only between treatments within the same time point.

### 3.4. Evaluation of Pot Experiments

A general enhancement in all measured plant growth parameters was evident in the pot trials. It was determined that both single-strain and consortium bacterial treatments significantly increased root and stem length, fresh and dry weight, and SPAD values. Plants treated with the A1 (isolate C-14), A2 (isolate C-27), and A3 (consortium) isolates had 5.8%, 9.5%, and 3.2% higher SPAD values, respectively, compared to the control plants in present study. The maximum SPAD value (47.2) was measured in barley seedlings treated with A2 treatment, while the lowest SPAD value was measured for the A4 (control) treatment (43.1) in the present study.

The PGPR isolates significantly affected the root and stem length of barley seedlings. According to the research results, the A2 treatment increased root length by 11.6% compared to the control treatment. The longest root (17.23 cm) was recorded in the A2 treatment. Although there was an increase in plant growth parameters in seeds treated with the consortium compared to the control, the longest stem (26.2 cm) and maximum fresh weight (60.03 mg) values were observed in the A2 treatment. Moreover, a significant increase in dry matter of barley seedlings was observed in response to PGPR isolates compared to the control. The maximum dry matter content was recorded in the A2 treatment (16.23 mg), while the minimum dry matter was observed in the A4 treatment (14.55 mg). It was determined that the A2 treatment exerted a more pronounced positive effect on plant growth compared to the other treatments. Details are presented in Table 4.

**Figure 4:**
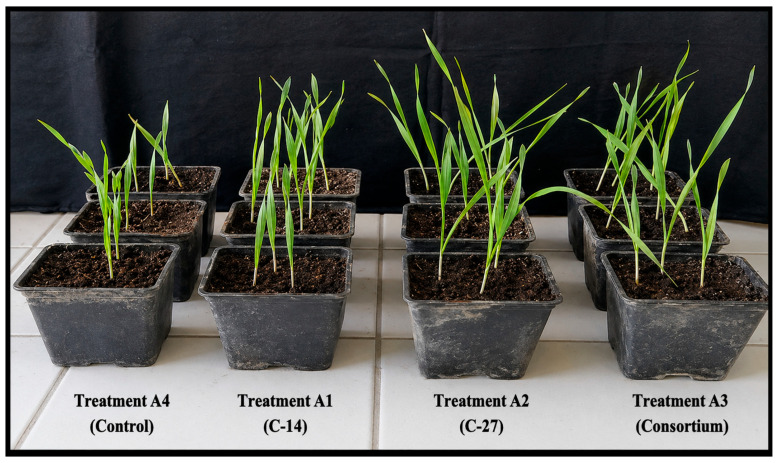
Barley seedling growth 30 days after inoculation with individual rhizobacterial isolates and a microbial consortium, compared with the uninoculated control.

**Figure 5:**
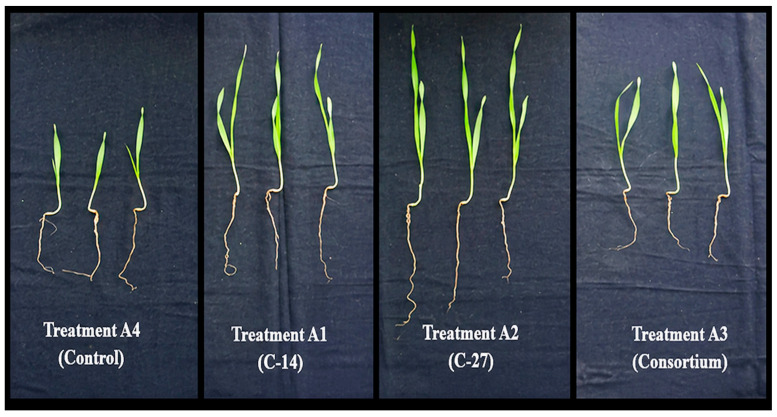
Barley seedling growth following inoculation with individual rhizobacterial isolates and a microbial consortium, compared with the uninoculated control.

The results of pot study showed that treatment of barley seeds with bacterial strains showed a positive effect on root and stem length (Figure 4 and Figure 5).

## 4. Discussion

The C-14 and C-27 isolates exhibited plant growth-promoting (PGP) traits, including indole compound production, over a 4-day period. Indole compounds are widely recognized bacterial metabolites associated with plant growth regulation and root development. These results suggest that the tested isolates possess potential PGP activity; however, their effects on plant growth parameters were evaluated separately under greenhouse conditions (Figure 4 and Figure 5). Bacteria have the ability to promote plant growth through the production and secretion of indole-derived compounds (IDCs). Indole-derived compounds, such as indole-3-acetic acid (IAA), enhance root development by stimulating primary root elongation as well as the proliferation of lateral and adventitious roots. These alterations in root architecture positively influence water absorption and nutrient uptake [26]. Many researchers have reported that endophyte bacteria such as *Bacillus* spp. found in the roots of different plants are good producers of IAA and that the concentration of synthesized bacterial indole-3-acetic acid (IAA) has a significant effect on the development of the root system [27,28]. Similar growth-promoting effects of IAA-producing *Bacillus* spp. have been widely reported. For instance, Solano-Alvarez et al. [29] reported that the application of phosphate-solubilizing and IAA-producing *B. cereus* isolated from the bean rhizosphere to tomato seeds increased root length and weight by 62% and 58%, respectively. Widowati et al. [30] observed that the inoculation of *B. subtilis* B11, an IAA-producing strain isolated from the roots, stems, and leaves of pepper, enhanced the stem length (19.75 cm) and root length (15.47 cm) of pepper seedlings. Similarly, there are also studies focusing on the effects of inoculation with IAA-producing *Bacillus* strains on barley plants. For instance Baris et al. [31] reported that inoculation of barley seeds with *Bacillus subtilis* and *B. megaterium*, which have the potential to produce IAA, increased grain yield and total yield by 15.1–27.8% and 14.5–18.5%, respectively. These results were in agreement with those reported by Hafez et al. [32] in barley plants. The current study confirms a significant positive relationship between root–stem length and IDC production. In the current greenhouse experiments, root lengths increased by 10, 18, and 8%, while shoot lengths increased by 60, 7%, and 43% after A1, A2, and A3 treatments, respectively (Table 4). Additionally, the treatment of the consortium may have had a lesser effect on root development compared to single-strain treatments, possibly due to negative interactions between the isolates that did not contribute positively to barley root elongation. Ma et al. [33] reported that single inoculation of barley seeds with *Bacillus mycoides* PM35, a strain possessing plant growth-promoting traits, resulted in significant increases in shoot length, root length, number of leaves, leaf area, shoot fresh weight, root fresh weight, and shoot dry weight compared to consortium-based treatments. A similar finding was reported by Anušauskas et al. [34], who stated that inoculation of barley with *Bacillus mycoides* enhanced plant growth parameters and, consequently, improved crop yield. To the best of our knowledge, although *B. tropicus* has been reported to enhance yield in crops such as wheat [35], rice [36], mung bean and mustard [37], and pineapple [38], this is the first study conducted on barley.

Consistent with the findings of our study, numerous reports indicate that, following inoculation, *Bacillus tropicus* can directly promote plant growth through the release of volatile organic compounds and the production of indole-derived metabolites [39,40]. Bourak et al. [41] reported that the application of *Bacillus tropicus* P4, a strain capable of synthesizing IAA (7.5 ± 0.3 μg mL^−1^), to bread wheat seeds significantly increased both root and shoot biomass. Similarly, Sanaullah et al. [42] reported that the application of wheat-derived biochar in combination with *Bacillus tropicus* increased spinach root biomass by 150% and root weight by a substantial 62.21% compared to the control. According to their findings, plant growth-promoting rhizobacteria (PGPR) enhance root biomass through the production of phytohormones, along with nutrient solubilization and the induction of systemic resistance. The application of PGPR likely improved nutrient use efficiency, thereby facilitating the development of a more extensive and vigorous root system. In this context, the observed increase in root weight is particularly important, as it contributes to overall plant health and resilience, especially under conditions of reduced nutrient availability. In our greenhouse study, barley treated with strain *Bacillus tropicus* C-27, with a high capacity for IDC production, clearly exhibited increased root biomass compared to the control.

SPAD is an indirect measure of the chlorophyll content in plant leaves. The treatment of PGPR in barley plants can enhance nitrogen uptake, resulting in increased leaf chlorophyll content. Purwanto and Suharti [43] reported a strong correlation between leaf chlorophyll content and nitrogen levels in leaves. According to Bagues et al. [44], bacterial inoculation significantly increases the growth of barley plants and SPAD values. For instance, Emami et al. [45] reported a significant increase in the SPAD value of barley plants inoculated with a salt-tolerant isolate possessing plant growth-promoting traits. Jamily et al. [46] stated that the inoculation of barley with *Bacillus subtilis*, which possesses the ability to produce indole-3-acetic acid (IAA), siderophores, and solubilize phosphate, increased the plant dry weight and SPAD value by approximately 10% to 20%, respectively. Similarly, Abideen et al. [47] reported that barley plants inoculated with endophytic *Pseudomonas* or *Pantoea* exhibited an approximately 18% increase in total chlorophyll content compared to the control plants. Similarly, El-Nagar et al. [13] reported that application of *Bacillus subtilis* and *Stenotrophomonas rhizophila* increased chlorophyll content in barley plants and significantly reduced the incidence of disease against *Pyrenophora teres*.

In the present study, both individual and consortium treatments led to an increase in total chlorophyll content in barley compared to the uninoculated control group. Moreover, the results showed that both single-strain and consortium PGPR treatments increased the SPAD value by 19% compared to the control group. The observed increase in chlorophyll content may be related to the ability of *Bacillus mycoides* C-14 and *Bacillus tropicus* C-27 to fix nitrogen in the soil more rapidly and efficiently, thereby providing the nitrogen necessary for growth, due to their high IDC production capacity. Additionally, consistent with our findings, Gul et al. [48] also reported enhanced chlorophyll content in barley plants inoculated with plant growth-promoting rhizobacteria (PGPR). In parallel with our findings, another study on single *Bacillus* inoculation of barley seeds by Bhambhu et al. [49] reported that the application of the IAA-producing *Bacillus subtilis* BREB 03, isolated from barley roots, significantly improved barley growth parameters. In a recent study, Ferioun et al. [50] reported that inoculation of barley seeds with *Pseudomonas amygdali*, which possesses IAA production and phosphate solubilization capabilities, increased the SPAD value by 12.9%. Consistent with these results, this study observed increases in root and stem length, root and stem dry weight, and chlorophyll content in plants treated with PGPR.

## 5. Conclusions

This study highlights the functional importance of endophytic *Bacillus* strains isolated from bell peppers (*Capsicum annuum* L.) in promoting barley plant growth. Molecular screening and phylogenetic analysis successfully identified the isolates as *Bacillus mycoides* C-14 *and Bacillus tropicus* C-27, each clustering within distinct, well-established taxonomic groups.

Comprehensive in vitro characterization revealed that these isolates exhibited plant growth-promoting (PGP) properties associated with IDC synthesis over a 4-day period. Future studies should include HPLC and spectral characterization methods with authentic standards to more accurately verify IAA production. The transition from laboratory assays to greenhouse trials successfully validated the real-world efficacy of these strains. While individual applications notably enhanced seedling vigor, it was the C-27 treatment that drove the most substantial improvements in root and shoot architecture, biomass accumulation, and chlorophyll content. These findings underscore the fact that synergistic interactions within a microbial community can surpass the limitations of single-strain inoculants, offering a more robust and multifaceted approach to plant growth promotion. These two strains are considered promising candidates for use as microbial fertilizers in agricultural applications due to their ability to produce IDC. However, further studies are needed to confirm the effectiveness of these endophytic bacteria in enhancing plant growth under actual field conditions.

## Figures and Tables

**Table 1:** Physiochemical characteristics of the garden soil used in the experiment.

Soil Characteristics	Value
ECe * (dS m^−1^)	2.6
Soil pH	5.3
Total N (µg kg^−1^)	13.1
Total P (µg kg^−1^)	3.98
Total K (µg kg^−1^)	18.2
Total OM (%)	0.84

* ECe, electrical conductivity of soil extract; N, nitrogen; P, phosphorus; K, potassium; OM, organic matter.

**Table 2:** Morphological and biochemical properties of the isolated endophytes.

Morphological and Biochemical Characterization	Isolates	
	*Bacillus mycoides* C-14	*Bacillus tropicus* C-27
Gram’s reaction	**+**	**+**
Endospore forming	**+**	**+**
Shape	Rods	Rods
Catalase	**+**	**+**
Oxidase	**+**	**−**
Motility	**+**	**+**
Endospore forming	**+**	**+**

Note: +, positive; −, negative.

**Table 3:** Molecular identification of endophytic bacterial isolates with their closely related species.

Isolate ID	NCBI Accession ID	Taxonomic Closest Relative	Percent Identity
C-14	PQ610418	*Bacillus mycoides*	99.93%
C-27	PQ610419	*Bacillus tropicus*	99.72%

**Table 4:** Height, weight, and chlorophyll content (SPAD units) (C) of barley plants (*H. vulgare* L.) in response to treatments with A1 (C-14), A2 (C-27) and A3 Consortium (C-14 + C-27) after 30 days.

Treatments	Root Length (cm) (Mean ± SD)	Stem Length (cm) (Mean ± SD)	Fresh Weight (mg) (Mean ± SD)		Dry Weight (mg) (Mean ± SD)		SPAD Units
			Root	Stem	Root	Stem	
A4 (Control)	14.6 ± 0.75 ^b,^*	15.03 ± 0.83 ^c^	39.5 ± 1.30 ^d^	86.7 ± 1.45 ^b^	3.14 ± 0.29 ^c^	14.55 ± 0.53 ^b^	45.6 ± 0.75 ^b^
A1 (C-14)	16.03 ± 0.73 ^a,b^	24.1 ± 0.91 ^a,b^	51.9 ± 2.02 ^b^	93.03 ± 0.75 ^a^	4.65 ± 0.46 ^b^	15.11 ± 0.29 ^ab^	47.2 ± 0.47 ^a^
A2 (C-27)	17.23 ± 1.02 ^a^	26.2 ± 1.47 ^a^	60.03 ± 1.45 ^a^	95.0 ± 1.30 ^a^	6.36 ± 0.42 ^a^	16.23 ± 0.62 ^a^	44.5 ± 0.42 ^a^
A3 Consortium (A1 + A2)	15.8 ± 0.62 ^a,b^	21.5 ± 0.88 ^b^	47.2 ± 0.45 ^c^	89.1 ± 0.43 ^b^	4.22 ± 0.37 ^b^	15.02 ± 0.9 ^b^	43.1 ± 0.4 ^b^

Note: A1 (C-14): *Bacillus mycoides*, A2 (C-27): *Bacillus tropicus*. A3 Consortium (C-14 + C-27). Each value represents the mean of three replicates. * Values sharing the same letter within a column are not statistically different at the 0.05 level of confidence.

## Data Availability

The original contributions presented in this study are included in the article. Further inquiries can be directed to the corresponding author.
